# A Review: Blue Fluorescent Zinc (II) Complexes for OLEDs—A Last Five-Year Recap

**DOI:** 10.3390/molecules28135272

**Published:** 2023-07-07

**Authors:** Thompho Jason Rashamuse, Reagan Lehlogonolo Mohlala, Elena Mabel Coyanis, Nomampondo Penelope Magwa

**Affiliations:** 1 Nanotechnology Innovation Centre, Health Platform, Advanced Materials Division, Mintek, Private Bag X3015, Randburg 2125, South Africa; reaganm@mintek.co.za (R.L.M.);; 2 Department of Chemistry, University of South Africa, Private Bag X6, Florida, Roodepoort 1710, South Africa

**Keywords:** OLEDS, blue Zn (II) complexes, thermal gravimetric, UV–vis absorption, photoluminescence

## Abstract

Blue emissions in organic light-emitting diodes (OLEDs) are essential for their application in solid-state lighting and full-colour flat panel displays. On the other hand, high-power blue emitters are still uncommon, especially those that can achieve the Commission Internationale de l’Eclairage (CIE, X, Y) coordinates of (0.14, 0.08) in the National Television System Committee (NTSC) blue standard and have high external quantum efficiencies (EQE) of more than 5% because their molecular design presents an enormous challenge. Therefore, creating effective, stable, pure, and deep blue fluorescent materials is vital. Here, it is addressed how useful blue fluorescent Zn (II) complexes are for making organic light-emitting diodes (OLEDs). Utilizing Zn (II) complexes is appealing because of their favourable luminous characteristics, acceptance and mobility, and affordability. This mini-review article aims to provide an overview of Zn (II) complexes that emit blue fluorescent light and have been reported since 2018, while highlighting the unique qualities that make them appropriate OLED materials.

## 1. Introduction

Metal complexes with small organic ligands have attracted a lot of attention in organic light-emitting diode (OLED) applications because of their exceptional electroluminescent properties [1,2,3,4,5,6,7,8,9,10]. OLEDs based on lower molecular weight molecules have become a potential replacement for LCD materials due to their numerous advantages, such as high efficiency, light weight, bright displays, and no required backlight. There are two main classes of light-emitting materials (LEDs), viz., fluorescent and phosphorescent materials, which are characterized by differences in the principle of light emission. Fluorescent materials emit light only in the singlet state, in contrast to phosphorescent materials, which can emit light in both the triplet and singlet states [11,12]. Blue fluorescent materials are essential in OLEDs, particularly those that can meet the Commission Internationale de l’Eclairage (CIE) coordinates of (0.14, 0.08) of the National Television System Committee (NTSC) standard blue while maintaining a high external quantum efficiency (EQE) of more than 5%. However, the majority of blue OLED materials continue to have issues, such as deprived stability, colour purity, a short operating lifespan, and high power consumption [13,14,15,16]. Because of the inherent difficulty in their molecular design, the development of deep blue materials with high efficiency is both fascinating and challenging. One of the remaining challenges in advancing OLED display devices is the development of compounds capable of generating blue fluorescent light without compromising purity, stability, or quantum efficiency. The luminescent properties of the complexes are determined by their electronic properties, the rigidity of the ligand binding, and the steric hindrance. The use of lanthanide and platinum group metal complexes has resulted from the need for highly efficient deep blue fluorescent emitters with narrow emission and photophysical behaviour [17,18]. Although the coordination compounds of lanthanides and platinoids have proven to be very stable and excellently bright during fabrication in OLEDs, the search for new and inexpensive sources of blue electroluminescence is of paramount importance given the high cost and low global reserves of these metal groups [19,20,21,22,23,24].

In recent years, great efforts have been made to develop highly blue organic zinc complexes as emitter and/or host materials for OLEDs and other applications such as latent fingerprints (LFPs), bioimaging, and biosensing [25,26,27,28,29,30,31,32]. The use of Zn (II) ions as the metal centre in the blue fluorescent design was motivated by their excellent luminescent properties, acceptability and transportability, and the low cost of Zn precursors. In addition, the high efficiency, thermal stability, and high solubility of the zinc complexes in most organic solvents make them suitable materials for the fabrication of OLEDs [33]. Another reason is that Zn complexes are often produced with a yield of 50–90%, in contrast to 10–25% for triscyclometalated iridium complexes, which are considered to be stable emitters for OLEDs [34,35]. Furthermore, it has been demonstrated that the optical characteristics of zinc emitters, as a four-coordinate metal, are identical to those of tris(8-hydroxyquinolinato)aluminium (Alq3), a key component of OLEDs [36]. Zinc salts such as zinc chloride (ZnCl_2_), zinc nitrate (Zn (NO_3_)_2_), zinc acetate hydrate (Zn (CH_3_CO_2_)_2_·2H_2_O), zinc acetate Zn (OAc)_2_, and zinc nitrate hexahydrate (Zn (NO_3_)_2_·6H_2_O are common sources of metal ions used in the coordination chemistry of Zn (II) complexes [37,38,39,40,41,42,43]. Lower-molecular-weight conjugated ligands derived from small organic molecules such as benzoxazole, benzothiazole, hydroxyquinoline, imidazoles, oxazoles, Schiff bases, etc. have been commonly used to generate zinc complexes [4,37,38,39,40,41,42,43]. In particular, the ligands with azomethine motifs have attracted the interest of blue electroluminescence researchers because of their strong fluorescence quantum yield and good temperature stability [35,36,37,38,39]. Furthermore, the electron transport ability and luminescence properties of the complexes can be easily tuned through ligand functionalization via the introduction of substituents with different electron donating/withdrawing abilities, making them promising active layer materials for OLED devices [35,36,37,38,39].

The main purpose of this review article to provide an overview of synthesis of various blue Zn (II) complexes as emitter and/or host materials, as well as their characterization, photodynamic parameters, and luminescence qualities that are important for the construction of OLED devices. This review includes articles published in the last five years from 2018 to 2023.

## 2. Results and Discussion

### 2.1. Blue Zn (II) Complexes as Emitters

**Scheme 1:**
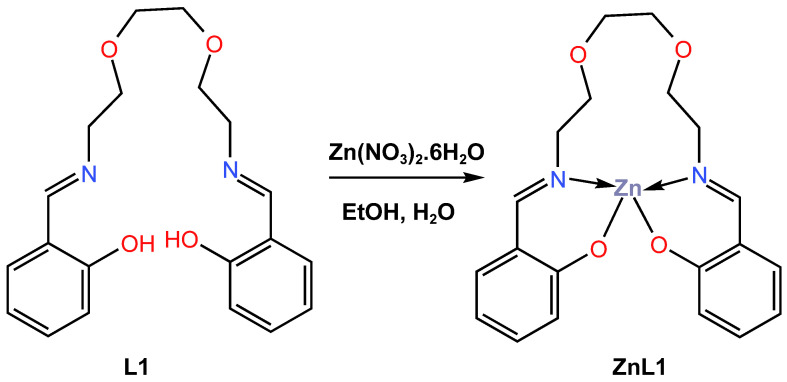
Molecular structure of ligand **L1** and its corresponding Zn (II) complex, **ZnL1**, reported by Kang et al. (2018) [43].

In 2018, Kang and co-workers [43] reported the quantum mechanical calculations and synthesis of a chemically stable blue-light-emitting Zn (II) complex, **Zn**L**1**, for OLED applications. The ZnL**1** complex was obtained as a white precipitate in a high yield of more than 95% from the reaction of *N*,*N*-*bis*(salicylidene)-3,6-dioxa1,8-diaminooctane **L**1 with zinc nitrate through a hydrolysis-free solution-based synthetic approach (Scheme 1). The differential thermal gravimetric analysis (TG-DTA) showed that the **ZnL1** complex is thermally stable at least up to 250 °C. The ultraviolet–visible (UV–Vis) absorption of the ligand **L1** and its **ZnL1** complex in both dichloromethane solution and powder form has been reported. The **ZnL1** complex showed three characteristic bands at 405, 315, and 275 nm in organic solution, while the two characteristic peaks at 355 and 270 nm corresponded to the **L1** ligand (see Table 1). The UV–Vis luminescence of the **L1** and **ZnL1** complexes in powder form, measured at 365 nm, showed that the **L1** produced no luminescence, whereas the **ZnL1** was reported to have a very strong blue emission with a maximum at 447 nm with quantum yield of 24.6%. The blue luminescence of **ZnL1** was also measured across the UV spectrum, with three bands identified at 390, 340, and 290 nm. The quantum mechanical calculations using density function theory (DFT) and Zerner’s Intermediate Neglect of Differential Overlap (ZINDO) were performed to obtain the equilibrium geometries. According to quantum mechanical calculations, the blue emission was attributed to an alteration in the chemical state of the ligand, which was instigated through coordination with Zn cations. The photoluminescence (PL) of the **ZnL1** complex in powder and dichloromethane solution, as well as in polymethyl methacrylate (PMMA) and cellulose acetate butyrate (CAB) hybrid films, was also studied. Compared to the powder form, it was observed that the corresponding CIE (x, y) coordinates of the organic solution and the PMMA hybrid film drifted near the red. The CIE coordinates (x, y) for the PMMA films were reported as 0.1707 and 0.1530, resulting in a change in the film’s PL colour from violet-blue to greenish-blue. In contrast, the incorporation of **ZnL1** into hybrid CAB films reveals that the CIE (x, y) coordinates vary from (0.1445, 0.1380) to (0.1619, 0.1881) depending on the molecular weight of **ZnL1**, which corresponds to a change in emission colour from deep blue to sky blue. In addition, the **ZnL1** powder was reported to have a lower quantum yield of 24%, which could be due to blue light emission from the inner particles not being fully scattered outward, compared to 43% for **ZnL1** in the organic solution phase. Furthermore, it has been reported that the incorporation of **ZnL1** in a PMMA film has a maximum quantum efficiency of up to 86% but suffers from poor photostability, while the incorporation of **ZnL1** in hybrid CAB thin films has a maximum quantum efficiency of 43.2% achieved with excellent photostability (see Table 2). According to the results, the reported incorporation of **ZnL1** in hybrid CAB film can be easily scaled and tuneable over a wide range for a variety of blue LED applications since the emission wavelength of the blue emission can be modified by adjusting the **ZnL1** content.

**Scheme 2:**
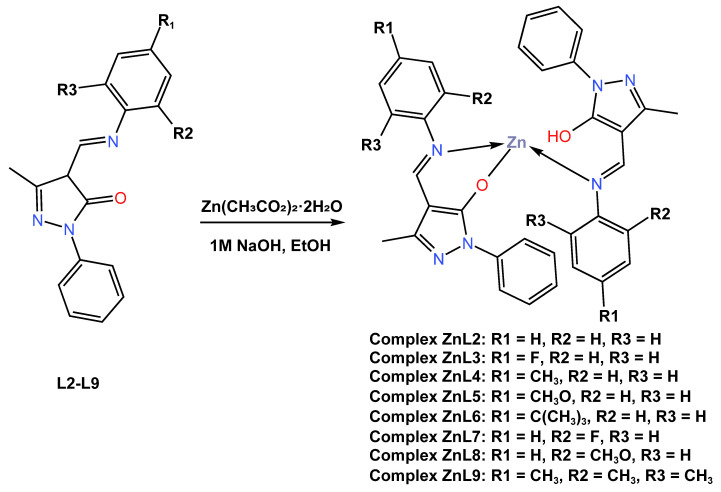
Molecular structure of ligands **L2**–**L9** and their corresponding Zn (II) complexes **ZnL2**–**ZnL9** reported by Gusev (2018) [50].

In the same year (2018), Gusev and co-workers [50] reported the synthesis of eight new tetrahedral blue fluorescent emitters Zn (II) complexes (**ZnL2**–**ZnL9**) based on 4-formylpyrazolone Schiff base derivatives **L2**–**L8** with yields ranging from 44 to 67 percent (Scheme 2). The complexes were constructed in a one-step procedure through the condensation of the 1-phenyl-3-methyl-4-formyl-5-pyrazolone with the corresponding aniline derivatives in the presence of zinc acetate dihydrate in a 2:2:1 M ratio. Crystal structure analysis showed complexes have a distorted tetrahedral geometry. It was further reported that the single crystal structures of complexes Zn**L2** and **ZnL7** crystallize in space group P1 of the triclinic system. The thermal stability of the complexes was determined to be between 90 and 200 °C, as determined by the thermogravimetric analysis (TGA) technique. It was reported that the UV–Vis absorption spectra of the **ZnL2**–**ZnL9** complexes recorded in a chloroform solution showed a prominent absorption band in the region of 340–352 nm, which was attributed to the superposition of ligand-based π*–π transitions of the azomethine backbone and intramolecular metal–ligand interactions within the whole assigned complex (see Table 1). The luminescent properties of these Zn (II) complexes in the solid state at room temperature have been reported to show strong luminescence with intense emission bands from 436 nm to 485 nm associated to ligand-centred π*–π transitions. It was further described that upon excitation at 395 nm, the quantum yields of the obtained complexes ranged from 9 to 55%, with complex **ZnL2** showing the highest quantum yield of 55%. All complexes in the solid state demonstrate a fine blue luminescence with CIE coordinates, alighting on the blue region with an x coordinate range between 0.158 and 0.26 and a y coordinate range of 0.27 and 0.288. The author also reported that excitation at 400–410 nm in chloroform solution gave intense emission bands with an emission maximum at 468–506 nm, with quantum yields smaller than those of solid samples and ranging from 0.6 to 2.9%. All of the complexes in the solid state displayed a subtle blue luminescence with CIE coordinates between 0.158 and 0.199 for the x coordinates and between 0.127 and 0.28 for the y coordinates, making them promising materials for building light-emitting diodes. Unfortunately, the authors did not report on the actual fabrication of complexes into devices.

**Scheme 3:**
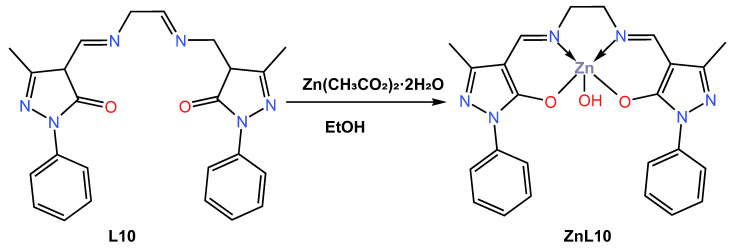
Molecular structure of ligand **L10** and its corresponding **ZnL10** complex reported by Gusev (2019) [44].

In 2019, Gusev et al. [44] reacted the 4-acylpyrazolone ligand **L10** ethylenediamine Schiff base with the Zn (II) ion in ethanol to give a five-coordinated blue emitter zinc **ZnL10** complex (Scheme 3). Slow evaporation of the ethanol solvent led to the formation of a crystal suitable for single X-ray crystallography, which was reported to crystallize in a monoclinic in space group P21/c, and the coordination polyhedron around the metal centre was reported to adopt a distorted square pyramidal. The TGA characterization of the complex showed that it has good thermal stability up to 153 °C. The absorption spectra of the anhydrous **L10**-based Zn (II) complex were investigated in tetrahydrofuran (THF), EtOH, MeCN, chloroform (CHCl_3_), and thin film. Upon irradiation with ultraviolet light at a wavelength corresponding to the maximum in the excitation spectrum (345–370 nm), a faint blue fluorescence was observed, with the maximum wavelength varying between 430 and 460 nm depending on the solvent polarity (see Table 1). Compared to the chloroform solution (460 nm vs. QY = 9.1%), the complex showed a deeper blue emission at 429 nm with a higher quantum yield (0.19%) in the more polar solvent acetonitrile. In addition, the author found that the complex exhibited deep blue fluorescence both in solution and in the solid state and was used to fabricate light-emitting devices (OLEDs). The **ZnL10** complex was evaluated in two OLED devices, a doped device with a configuration of ITO|TPD|**ZnL10**: mCP|PDB|Ca|Al and an undoped device with a configuration of ITO|TPD|**ZnL10**|PDB|Ca|Al. The undoped device was found to have a turn-on voltage of 7.5 V, a maximum brightness of 13,000 cd/m^2^ (10 V), and a maximum current efficiency of 8.6 cd/A (see Table 2). While doped devices had a lower turn-on voltage of 5 volts, a maximum brightness of 17,000 cd/m^2^ at a voltage of 14 volts, and a maximum current yield of 13.8 cd/A, the doped OLED was found to have an external quantum efficiency of almost 5% with CIE coordinates of 0.22 and 0.19, which lags behind the 2.9% EQE for the undoped OLED with a CIE value of 0.24 and 0.27.

**Scheme 4:**
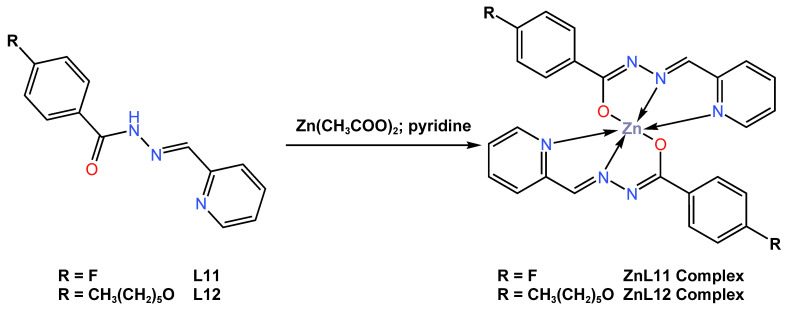
Molecular structure of ligands **L11** and **L12**, as well as their corresponding Zn (II) complexes **ZnL11** and **ZnL12**, reported by Diana et al. (2019) [51].

In the same year of 2019, Diana and colleagues [51] reported the synthesis of two new blue fluorescent emitter octahedral complexes, **ZnL11** and **ZnL12**, with yields of 80 and 70%, respectively. The complexes were obtained from the reaction of Zn (II) acetate with Schiff-based ligands, either 4-fluoro-*N*-(pyridin-2-ylmethylene)benzohydrazide **L11** or 4-(hexyloxy)-*N*-(pyridin-2-ylmethylene)benzohydrazide **L12**, in a 2:1 stoichiometric ratio in pyridine (Scheme 4). It was reported that the complex crystallized in the triclinic P-1 space group and assumed a deformed octahedral geometry around the Zn atom. Photophysical measurements of the complexes were investigated in dichloromethane or on spin-coated quartz slides. The UV–Vis absorption of both complexes in dichloromethane solution was reported to be at maximum wavelengths of 390 nm for **ZnL11** and 382 nm for **ZnL12**, while the complexes in the thin-film state exhibited UV–Vis absorption at maximum wavelengths of 335 nm and 351 nm for **ZnL11** and **ZnL12** (see Table 1). The quantum yields of **ZnL11** and **ZnL12** in the thin film state were reported to be 23 and 26%, respectively. The photoluminescence (PL) properties of the **ZnL11 and ZnL12** complexes were reported to exhibit intense blue emission in solid state at 473 and 472 nm, while, in diluted solution, the complexes were found to be poor emitters, ascribed to their aggregation-induced emission (AIE) property. The authors suggested that the data obtained makes the complexes very promising as easy and low-cost fluorophore dopants for blue emissive layers. Unfortunately, the authors did not report on the fabrication of the complexes into devices.

**Scheme 5:**
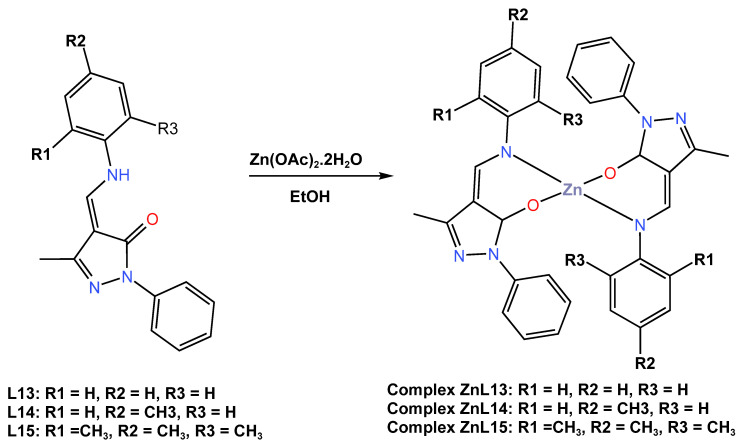
Molecular structure of ligands **L13**–**L15** and their corresponding Zn (II) complexes **ZnL13**–**ZnL15** reported by Gusev et al. (2021) [45].

In 2021, Gusev and others [45] described a comprehensive investigation of the molecular structure, thermal stability, and electrochemical properties of three blue emitter Zn (II) complexes, **ZnL13**, **ZnL14**, and **ZnL13**, based on 1-phenyl-3-methyl-4-formyl-5-pyrazolone azomethine ligands, as shown in Scheme 5. The applicability of these Zn (II) complexes as an active layer in light-emitting diodes was investigated using numerous physical–chemical methods. The authors previously reported the crystal structures of **ZnL13** and **ZnL14** in 2018 and 2019, respectively [42,43]. However, it was discovered that the new crystal structure of **ZnL15** is triclinic, with the zinc ion crystallizing in a tetradentate geometry with the Schiff base ligands and having a distorted tetrahedral geometry. Both complexes showed high thermal stability as estimated by decomposition temperatures, with a 2% weight loss at 330 °C for **ZnL15**, 335 °C for **ZnL14**, and 355 °C for **ZnL13**. The electrochemical performance of the Zn (II) complexes investigated in dry DMF by cyclic voltammetry measurements in a three-electrode cell system revealed irreversible oxidation with a peak maximum at 0.93–1.05 V, while the reduction process established quasi-reversible recovery peaks with recovery initiation potential detected in the range of −0.44 to −0.65 V. The energies of the highest energy occupied molecular orbital (HOMO) of these three complexes were estimated to be between −5.21 and 5.33 eV, while their lowest energy unoccupied molecular orbital (LUMO) energy values were found to be located in the range of −1.87 to −3.25 eV. In DMF solution, all complexes showed UV–Vis absorption at maximum wavelengths of 339 nm for **ZnL13**, 344 nm for **ZnL14**, and 307 nm for **ZnL15**, while both complexes showed emission bands with maxima at 468, 459, and 436 nm, with quantum yields up to 12% in a blue region (see Table 1). In the solid state, both complexes were reported to have emission bands in the visible blue region with maxima at 470, 466, and 438 nm for the complexes of zinc ions coordinated with **L13**, **L14**, and **L15** ligands, respectively, and afforded quantum yields of up to 55%. Engineered light-emitting diodes of all three complexes in devices with the configuration of ITO|CuI|CzNC|**ZnL13**, **ZnL14**, or **ZnL15**|TSPO1|TPBi|Ca|Al showed that only devices from the complex **ZnL15** exhibited the deepest blue emission, with an external quantum efficiency of 2, 5% at CIE coordinates (X, Y) of 0.24; 0.28, with a maximum brightness of 11,600 cd/m^2^, and with a maximum current efficiency of 4.3 cd/A at a low input voltage of 4.0 V (see Table 2).

**Scheme 6:**
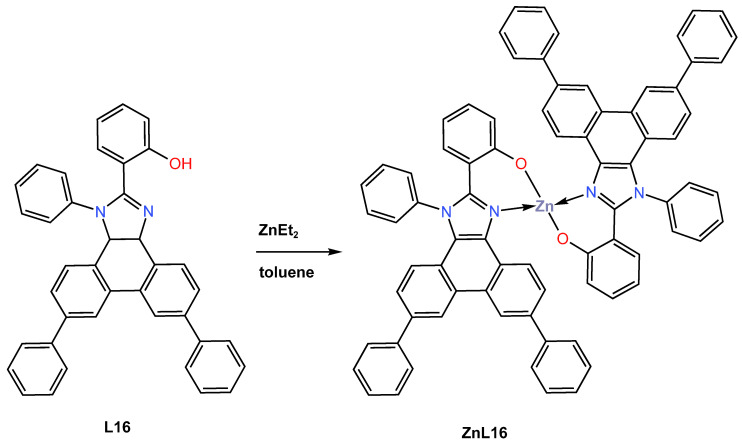
Molecular structure of ligand **L16** and its corresponding Zn (II) complex **ZnL16** reported by Yu (2021) [46].

In the same year, 2021, Yu et al. [46] described the design and synthesis of a new blue fluorescent zinc complex **ZnL16** obtained in 75% yield from the coordination reaction of diethylzinc with 2-(1,6,9-triphenyl-1*H-*phenanthro[9,10-d] imidazole-2-yl)phenol (**L16**) as an organic ligand in toluene under an inert atmosphere condition (Scheme 6). **ZnL16** was reported to exhibit high thermal stability, as determined by TGA, with a decomposition temperature corresponding to 5% weight loss at 495 °C and no glass transition as determined by DSC. The electrochemical properties of **ZnL16** were probed through CV measurement in DMF and reported to display irreversible oxidation with a HOMO energy level of −5.33 eV, close to those of common hole-transporting materials. The charge-transporting ability of **ZnL16** was fabricated and investigated in two devices with a configuration of ITO|Zn(TPPI)_2_|TAPC|Al for hole-only and ITO|TmPyPB|Zn(TPPI)_2_|LiF|Al for electron-only. The author reported that the TAPC-based device has a higher LUMO of −2.0 eV, resulting in a large electron injection barrier compared to the LUMO of −4.3 eV of an aluminium cathode. In contrast, the hole-blocking material TmPyPB has a HOMO of −6.7 eV, which could inhibit hole injection of ITO with a HOMO of −4.8 eV to give the device a pure electron current. According to the space charge limited current (SCLC) method, the hole and electron mobilities of **ZnL16** were computed to be 2.46 9 10^−11^ cm^2^ V^−1^ s^−1^ and 2.61 9 10^−11^ cm^2^ V^−1^ s^−1^, respectively. These characteristics signify that **ZnL16** is a hole-type material. The authors further investigated the photophysical characteristics of **ZnL16** in dichloromethane solution and neat film using UV–Vis adsorption and photoluminescence (PL) spectra at ambient and low temperatures. According to UV–vis adsorption, the complex was reported to show three absorption bands at 275, 342, and 376 nm attributed to the benzene rings, the π-π* transition of phenanthro[9,10-*d*]imidazole, and the delocalized π-π* transition from the 2-substituted phenolate to the phenanthro[9,10-*d*]imidazole ring, respectively (see Table 1). The photoluminescence (PL) properties of the **ZnL16** complex were reported to exhibit blue emission in both dichloromethane solution (at 467, 473 nm) and film state (447 nm) at room temperature with quantum yields 68% (in solution) and 21% (thin film). The authors further reported that the preparation of the **ZNL16** complex with a configuration of ITO|HATCN|TAPC|TCTA|**ZnL16**|TmPyPB|LiF|Al provides good blue emission in an undoped device with a maximum current efficiency of 3.79 cd/A at a low input voltage of 3 V, a maximum power efficiency of 3.97 lm/W, and a maximum brightness of 1153 cd/m^2^, with an external quantum efficiency of 2.47% at CIE coordinates (X, Y) of 0.17 and 0.18. The data obtained recommended **ZnL16** as a good blue emitter and host material (see Table 2).

**Scheme 7:**
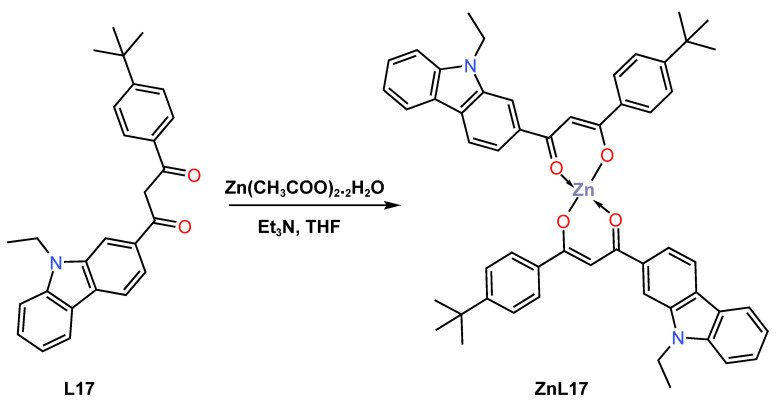
Molecular structure of **L17** and **ZnL17** complex reported by Liu (2021) [47].

Also in 2021, Liu and colleagues [47] reported efficient deep blue-emitting material electroluminescent devices based on a new β-diketone Zn (II) complex **ZnL17** for application in OLEDs. The complex was prepared as a yellow powder in 70% yield from the reaction of 1-(4-(*tert*-butyl)phenyl)-3-(9-ethyl-9H-carbazol-3-yl)propan-1,3-dione **L17** with zinc acetate dihydrate (THF) using redistilled triethylamine (Et_3_N) in tetrahydrofuran solvent (Scheme 7). The mechanism of luminescence was also explored by DFT and time-dependent DFT calculations, which revealed the zinc complex had a fluorescence emission mechanism of **ZnL17** that switched from LUMO to HOMO, with the former being mainly on the phenyl group and the latter being mainly on the carbazole moiety. The calculated energy-level gap of HOMO and LUMO was reported to be 3.29 eV, while the optical gap was 2.95 eV. It was also established that the complex was thermally stable up to 327 °C, as determined by the TGA studies. The UV–Vis absorption of **ZnL17** in dichloromethane has been reported in the range 200–400 nm, with absorption peaks at 240, 285, 331, and 384 nm devising from π-π* transitions, while the long-wave absorptions at 331 and 384 nm were assigned to a large conjugated system of carbazolyl units (see Table 1). The photoluminescent property of the complex was observed to have emission band at 453 nm with 5.5% in dichloromethane solution. The author described the manufacture of three devices with an ITO|HAT-CN|NPB|MADN:**ZnL17**|TPBi(40 nm)/LiF(1 nm)|Al configuration utilizing three distinct guest-doping concentrations of 1 weight (wt)%, 3 wt%, and 5 wt% of **ZnL17** complex. The 3% doping device demonstrated deep-blue electroluminescence and attained the greatest electroluminescent performances among them. At maximum voltage of 8 V, this deep-blue device produced a CIE value of (0.1558, 0.0901), which is quite near to the NTSC standard blue value (0.14, 0.08) (see Table 2). The 3 wt% doping device achieved the best electroluminescent performances with maximum current efficiency of 1.63 cd/A, external quantum efficiency of 1.54%, power efficiency of 1.5 lm/W, and maximum brightness of 2096 cd/m^2^. Despite the fact that the 3 wt% device had a deep-blue emission and a CIE Y coordinate value similar to the NTSC standard blue value, improving its structure is required due to the use of maximum voltage to achieve maximum brightness.

**Scheme 8:**
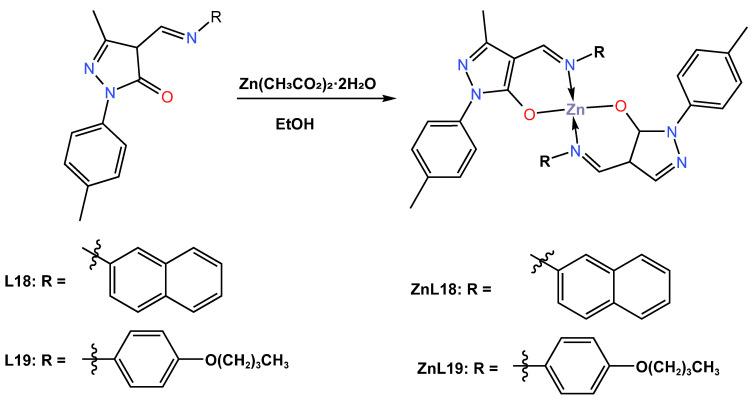
Molecular structure of ligands (**L18** and **L19**) and their corresponding Zn (II) complexes **ZnL18** and **ZnL19** reported by Solanki (2022) [48].

In 2022, Solanki et al. [48] synthesized two blue emitter Zn (II) complexes (**ZnL18** and **ZnL19**) with pyrazolone-based azomethine ligands and used them for OLED applications. The reaction of the ligands 5-methyl-4-((naphthalene-1-ylimino)methyl)-2-(p-tolyl)-2,4-dihydro-3*H*-pyrazol-3-one **(L18**) and 5-methyl-4-((4-Butoxyphenyl)imino)methyl)-2-(p-tolyl)-2,4-dihydro-3***H****-*pyrazol-3-one (**L19**) with zinc acetate dihydrate in an ethanol solution followed by recrystallization from ethanol: methylene chloride (2:1) provided tetradentate Zn (II) complexes **ZnL18** and **ZnL19** in overall yields of 70 and 80%, respectively (Scheme 8). Only the **ZnL18** complex was examined using single crystal X-ray diffraction and was found to crystallize in the triclinic P-1 space group in ethanol: methylene chloride (ratio 2:1). Both complexes were reported to have effective thermal stability at high decomposition temperatures greater than 350 °C, and both complexes were amorphous in nature with no glass transition temperature, as measured by TGA and DSC. The UV–Vis absorption and photoluminescence properties of **ZnL18** and **ZnL19** were reported both in DMF solution and in the solid state at room temperature. The redox potential of Zn (II) complexes measured by the cyclic voltammetry analysis was reported to have HOMO energy values of −5.54 and −5.46 eV and LUMO energy levels of −2.73 and −2.60 eV. The UV–Vis absorption of **ZnL18** and **ZnL19** in solutions was found to emit a blue emission at 424 and 432 nm, respectively, while in the solid state, a red shift emission at 466 and 468 nm for **ZnL18** and **ZnL19** was observed (see Table 1). The quantum yields of 12.1% for **ZnL18** and 4.2% for **ZnL19** were reported in DMF solution, whereas they were 5.7 and 1.9% for **ZnL18** and **ZnL19** in the solid state, respectively. Furthermore, the author reported that these complexes showed subtle blue photoluminescence in solution and in the solid state, potentially making them suitable materials for OLED fabrication. Both Zn (II) complexes were further processed in solution with doped and undoped devices that constructed light-emitting diodes in a multilayer architecture with device configurations of ITO|PEDOT:PSS|(CBP|**ZnL18** or **ZnL19**|TPBi|LiF|Al, and their electroluminescence was determined. The authors reported that the **ZnL18** complex-based device had a maximum current efficiency of 0.9 cd/A at a lower turn-on voltage of 3.5 V, an external quantum efficiency of 3.2%, a maximum brightness of 868 cd/m^2^, and a power efficiency of 0.4 lm/W (see Table 2). On the other hand, the solution-processed, device-based **ZnL19** complex was reported to have a maximum current efficiency of 0.4 cd/A at a lower turn-on voltage of 5 V, an external quantum efficiency of 1.4%, a maximum brightness of 664 cd/m^2^, and an energy efficiency of 0.2 lm/w (see Table 2). The CIE value of a **ZnL18** complex-based device was found to exhibit deep blue emission at 0.16 for the x-coordinate and a remarkable 0.09 for the y-coordinate, while (0.18, 0.11) coordinates were recorded for a **ZnL19** complex-based device. The deep blue emission exhibited by the **ZnL19** complex is aligned with the NTSC colour coordinates, meeting the high colour saturation requirements in display technology.

### 2.2. Blue Zn (II) Complexes as Host Materials

**Scheme 9:**
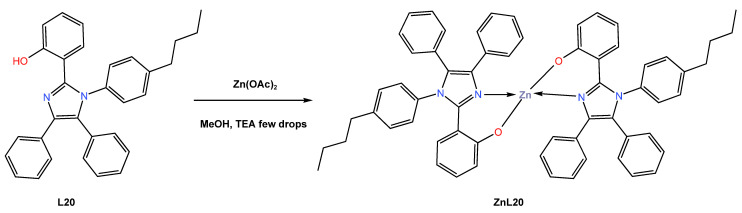
Molecular structure of ligand **L20** and its corresponding Zn (II) complex **ZnL20** reported by Kempegowda (2021) [49].

In 2021, Kempegowda and co-workers [49] reported the synthesis of the novel blue-luminescent Zn (II) coordination complex **ZnL20**, which is indispensable in OLEDs. Applications of the **ZnL20** complex, such as anti-counterfeiting and latent finger printing (LFP), have also been described due to the strong cyan-blue luminescence emission with high quantum efficiency. The **ZnL17** complex was reported to be synthesized as a white crystalline solid from the reaction of the bisimidazolyl phenol (**L20**) ligand with zinc acetate using methanol as a solvent (Scheme 9). The complex **ZnL20** was found to crystallize in a monoclinic crystal system in the C2/c space group, containing a molecule in an asymmetric unit. Because of the complex’s great thermal stability up to 472 °C, the authors proposed that it can be used to make thin, homogeneous films through thermal evaporation that are suitable for the manufacturing of OLEDs and host materials. The authors reported that absorption and emission of **L20** and **ZnL20** molecules were red-shifted with increasing solvent polarity, with the maximums cantered at 332 and 322 nm, respectively. The shift in absorption could possibly be attributed to the possible π-π* transition. Authors also reported that the fluorescence quantum yield increases with solvent polarity (from 0.561% in benzene to 0.639% in DMSO). According to DFT calculations, the first absorption maximum occurs at about 344 nm, which corresponds to the S_0_-S_1_ transition (see Table 1). The assembled light-emitting diodes of the **ZnL20** complex in an undoped multilayer OLED device with a configuration of ITO|NPD|CBP|**ZnL120**:TPBi|LiF|Al displayed good electroluminescent properties, with a maximum brightness of 13,500 cd/m^2^ and a maximum EQE of 3.8% (see Table 2). It was further observed that the solid-state **ZnL20** emits strong cyan-blue luminescence with a high quantum yield of 0.83, while the CIE of the complex showed the colour coordinates for the device at x = 0.1468 and y = 0. Due to the high quantum yield, better absorption, high thermal stability, and high emissivity of the **ZnL20** complex, the study was extended for possible applications of the complex as a fluorescent dye in latent fingerprint (LFP) technology. It was reported that LFPs can be better visualized using **ZnL20** under UV light as the standard reference material, indicating that the **ZnL20** produced is considered to be an excellent means of visualizing LFPs. The authors demonstrated the utilization of Zn-BDI as an emissive material to construct efficient, high-luminance blue OLEDs.

## 3. Conclusions

OLEDs have made remarkable strides in the last decade, driven by the massive flat panel and solid-state lighting markets. Recent research efforts in OLED materials have focused on developing efficient, stable, and pure blue emitters to capture the much higher performance of lower-energy colours. Researchers have been able to overcome hurdles in modifying the structure and electronic properties of Zn (II) complexes, which has had a substantial impact on their photophysical properties thanks to the discovery of new synthetic pathways. The creation of efficient and resilient blue emitters for OLED applications is made possible by careful ligand selection. The perfect candidates for Zn (II) complexes for commercial applications should show high stability in devices with more than 90% PLQYs and CIE coordinate values (X,Y) conforming to the values of (0.14, 0.08), corresponding to the Internationale de l’Eclairage in the National Standard Blue Standard of the Television System Committee (NTSC). Such ideal Zn (II) complex candidates have been described in this review, and one of them has been reported by Solanki and co-workers [48]. In addition, the high stability of blue Zn (II) complexes was demonstrated by Kempegowda and co-workers [49]. These kinds of discoveries of novel Zn (II) complexes set the foundation for the future development of complexes based on fundamental molecular frameworks.

In the future, understanding the relationship between structure, electrical properties, and other photophysical aspects will be crucial for the development of next-generation Zn (II) complexes for OLEDs. In addition, the standardization of photophysical data would help to understand the complex systems already described.

## Figures and Tables

**Table 1:** An overview of the photophysical properties of reported blue Zn (II) complexes as emitters and hosts materials from 2018 to 2023.

Complexes	Absorption λ_max_ (nm)	Solvent	Emission				Ref.
			Solution		Solid		
			*Λ*max (nm)	QY (%)	*λ*max (nm)	*λ*max (nm)	
**ZnL1**	275, 315, 405	Dichloromethane	290, 340, 390	42.6	447	24.6	[41]
**ZnL2**	340	Chloroform	468	1.4	471	55.3	[42]
**ZnL3**	338	Chloroform	480	2.7	480	41.7	[42]
**ZnL4**	344	Chloroform	471	1.5	485	17.7	[42]
**ZnL5**	352	Chloroform	467	2.3	485	9.6	[42]
**ZnL6**	341	Chloroform	474	1.6	464	16.9	[42]
**ZnL7**	339	Chloroform	480	2.9	464	48.7	[42]
**ZnL8**	337	Chloroform	470	0.6	473	10.5	[42]
**ZnL9**	339	Chloroform	481, 506	2.1	436	54.5	[42]
**ZnL10**		Acetonitrile	429	19	419, 433, 468	5.1	[44]
	315	Chloroform	460	9.1			
		Ethanol	432	9.3			
	330	Tetrahydrofuran	441	6.1			
**ZnL11**	390	Dichloromethane	507	-	473	23	
**ZnL12**	382	Dichloromethane	501	-	472	26	
**ZnL13**	339	Dimethylformamide	468,	6.7	470	55	[45]
**ZnL14**	344	Dimethylformamide	446, 459	7.9	445, 466	29	[45]
**ZnL15**	307	Dimethylformamide	436	12.1	438, 458	54	[45]
**ZnL16**	275, 342,376	Dichloromethane	467, 473	68	447	21	[46]
**ZnL17**	240, 285, 331,384	Dichloromethane	453	5.5	-	-	[47]
**ZnL18**	270, 350, 389	Dimethylformamide	424	12.1	466	5.7	[48]
**ZnL19**	270, 348, 390	Dimethylformamide	432	4.2	468	1.9	[48]
**ZnL20**	313	Benzene	411	0.561	-	-	[49]
	314	Toluene	413	0.585	-	-	
	316	Tetrahydrofuran	459	0.586	-	-	
	316	Ethyl acetate	459	0.589	-	-	
	317	Dichloromethane	463	0.603	-	-	
	319	Dioxane	470	0.609	-	-	
	321	Methanol	477	0.632	-	-	
	322	Ethanol	479	0.635	-	-	
	322	Acetonitrile	481	0.631	-	-	
	328	Dimethylsulfoxide	483	0.639	-	-	

λ_max_: Maximum wavelength; QY: Quantum yield; dash (-): not reported.

**Table 2:** An overview of the electroluminescent properties of reported Zn (II) complexes as emitters and hosts materials from 2018 to 2023.

Complex	Device Structure	Max. Current Efficiency, cd/A	Max. Power Efficiency, lm/W	CIE Coordinates (x; y)	Max. Brightness, cd/m^2^	Max. EQE, %	Ref.
**Blue Zn (II) Complexes as emitters**							
**ZnL1**	**ZnL1**-CAB hybrid polymer films	-	-	(0.145–0.1619; 0.1380–12,881)	-	43	[41]
	**ZnL1**-CAB hybrid polymer films			(0.1707, 0.1530)		86	
**ZnL10**	ITO|TPD|**ZnL10**:mCP|PDB|Ca|Al	8.6	2.6	(0.24; 0.27)	13,000 cd/m^2^ at 10 V	2.9	[44]
	ITO|TPD|**ZnL10**|PDB|Ca|Al	13.8	7.6		17,000 cd/m^2^ at 14 V	5.0	
**ZnL13**	ITO|CuI|CzNC|**ZnL13**|TSPO1|TPBi|Ca|Al	4.2	1.8	(0.21; 0.29)	5550 cd/m^2^ at 4.0 V	2.1	[45]
**ZnL14**	ITO|CuI|CzNC|**ZnL14**|TSPO1|TPBi|Ca|Al	6.6	2.9	(0.18; 0.28)	4300 cd/m^2^ at 3.2 V	3.3	[45]
**ZnL15**	ITO|CuI|CzNC|**ZnL15**|TSPO1|TPBi|Ca|Al	4.3	1.8	(0.24; 0.28)	11,600 cd/m^2^ at 4.0 V	2.5	[45]
**ZnL16**	ITO|HATCN|TAPC|TCTA|**ZnL16**|TmPyPB|LiF/Al	3.79	3.97	(0.17; 0.18)	1153 cd/m^2^ at 3 V	2.47	[46]
**ZnL17**	ITO|HAT-CN|NPB|MADN:**ZnL17**/TPBi|LiF|Al	1.63	1.50	(0.1558; 0.0901)	2096 cd/m^2^ at 8 V	1.54	[47]
**ZnL18**	ITO|PEDOT:PSS|(CBP|**ZnL18**|TPBi|LiF|Al	0.9	0.4	(0.16; 0.09)	868 cd/m^2^ at 3.5 V	3.2	[48]
**ZnL19**	ITO|PEDOT:PSS|(CBP|**ZnL19**|TPBi|LiF|Al	0.4	0.2	(0.18; 0.11)	664 cd/m^2^ at 5 V	1.4	[48]
**Blue Zn (II) Complexes as host material**							
**ZnL20**	ITO|NPD|CBP|**ZnL120**:TPBi|LiF|Al	5.9	0.83	(0.1468; 0.1903)	13500 cd/m^2^ at 16 V	3.8	[49]

Dash (-) means the values were not reported by the authors.

## Data Availability

Data supporting reported review appear in the manuscript.
